# Association Between DRD2 and DRD4 Polymorphisms and Eating Disorders in an Italian Population

**DOI:** 10.3389/fnut.2022.838177

**Published:** 2022-03-14

**Authors:** Maria Rachele Ceccarini, Simona Fittipaldi, Cinzia Ciccacci, Erika Granese, Federica Centofanti, Laura Dalla Ragione, Matteo Bertelli, Tommaso Beccari, Annalisa Botta

**Affiliations:** ^1^Department of Pharmaceutical Science, University of Perugia, Perugia, Italy; ^2^Consorzio Interuniversitario per le Biotecnologie (C.I.B), Trieste, Italy; ^3^Department of Biomedicine and Prevention, University of Rome Tor Vergata, Rome, Italy; ^4^UniCamillus, Saint Camillus International University of Health Sciences, Rome, Italy; ^5^Food Science and Human Nutrition Unit, University Campus Biomedico of Rome, Rome, Italy; ^6^MAGI Euregio, Bolzano, Italy; ^7^EBTNA-Lab, Rovereto, Italy

**Keywords:** eating disorders, anorexia nervosa, bulimia nervosa, binge eating, *DRD2*, *DRD4*, *ANKK1*, genetic polymorphisms

## Abstract

Anorexia nervosa (AN), bulimia nervosa (BN), and binge eating disorder (BED) are the three most common eating disorders (EDs). Their etiopathogenesis is multifactorial where both the environmental and genetic factors contribute to the disease outcome and severity. Several polymorphisms in genes involved in the dopaminergic pathways seem to be relevant in the susceptibility to EDs, but their role has not been fully elucidated yet. In this study, we have analyzed the association between selected common polymorphisms in the *DRD2* and *DRD4* genes in a large cohort of Italian patients affected by AN (*n* = 332), BN (*n* = 122), and BED (*n* = 132) compared to healthy controls (CTRs) (*n* = 172). Allelic and genotypic frequencies have been also correlated with the main psychopathological and clinical comorbidities often observed in patients. Our results showed significant associations of the *DRD2*-rs6277 single nucleotide polymorphism (SNP) with AN and BN, of the *DRD4*-rs936461 SNP with BN and BED and of *DRD4* 120-bp tandem repeat (TR) polymorphism (SS plus LS genotypes) with BED susceptibility. Moreover, genotyping of *DRD4* 48-bp variable number TR (VNTR) identified the presence of ≥7R alleles as risk factors to develop each type of EDs. The study also showed that ED subjects with a history of drugs abuse were characterized by a significantly higher frequency of the *DRD4* rs1800955 TT genotype and *DRD4* 120-bp TR short-allele. Our findings suggest that specific combinations of variants in the *DRD2* and *DRD4* genes are predisposing factors not only for EDs but also for some psychopathological features often coupled specifically to AN, BN, and BED. Further functional research studies are needed to better clarify the complex role of these proteins and to develop novel therapeutic compounds based on dopamine modulation.

## Introduction

Eating disorders (EDs), divided into eight categories according to the *Diagnostic and Statistical Manual of Mental Disorders Fifth Edition* (DSM-V) (1), are serious psychiatric illnesses and they have been increased over the past 50 years (2). Anorexia nervosa (AN), bulimia nervosa (BN), and binge eating disorder (BED) are the three most common severely debilitating disorders with high morbidity and mortality rates among adolescents (3). Unfortunately, there is still much to be learned about EDs: their etiopathogenesis, their development, their treatment (4), and more importantly, their genetic background are crucial points to be clarified (5). Twin, family, and adoption studies have shown that EDs are heritable (6). Different and independent research works demonstrated that the risk of developing AN is ~11-fold greater for a first-degree relative of individuals with AN or BN than for the general population (7, 8). The relative risk for BN is around 4 times greater for female relatives of affected probands (7, 8). Moreover, twin studies estimated a hereditability between 56 and 74% for AN (9, 10), 47% for BN (11), and between 41 and 57% for BED (12, 13). There are also numerous evidence regarding the multi-factorial nature of EDs where environmental factors, psychological and traumatic conditions as well as epigenetic mechanisms contribute to the disease etiopathogenesis (2). The polygenic component of EDs susceptibility has been thoroughly investigated over the past 30 years with candidate genes association studies and, more recently, using genome-wide association approaches (GWAS), which identified several susceptibility loci (14–17). Among the identified pathways, many papers have focused on the dopaminergic system genes (18–20), but their association with EDs has not been yet fully elucidated. Dopamine (DA) is a catecholamine neurotransmitter highly expressed in the central nervous system (CNS) and implicated in a wide array of brain functions, including those regulating food intake (21). Malfunction of the DA system has been associated with a number of psychiatric illnesses, such as schizophrenia (22, 23), maniac depressive, and attention-deficit/hyperactivity disorders (24, 25). In the hypothalamus, DA release is associated with the duration of meal consumption and dysfunction of DA receptors has been suggested to predispose to EDs by altering the feeding behavior (26), by disturbing reward associated with food intake (27), and by distorting of body shape and image (28). Based on these evidences, genes coding for enzymes, receptors, and transporters involved in DA pathways have been considered crucial candidates for genetic association studies in patients affected by EDs, including AN, BN, and BED. DA acts by binding five different dopamine receptors (DRDs) that are members of G protein-coupled receptors divided into two major families: the D1-like family (DRD1 and DRD5) and the D2-like family (DRD2, DRD3, and DRD4). The first family D1-like receptors activate the adenylyl cyclase and increase the second messenger cyclic adenosine monophosphate (cAMP), while the D2-family inhibits the adenylyl cyclase reducing cAMP (29, 30). The *DRD2* gene (MIM#126450), localized on chromosome 11 q22-q23, encodes for a seven-transmembrane G protein-linked receptor that binds DA (31). This receptor has been extensively associated with drug addiction, alcoholism, substance abuse (32). The expression of *DRD2* is regulated *in cis* by the adjacent ankyrin repeat and kinase domain containing 1 (*ANKK1*, MIM#608774) gene, located proximal to *DRD2* (33). Both the *DRD2* and *ANKK1* genes have been significantly associated with EDs in several studies (27, 34–36). In particular, a specific single nucleotide polymorphism (SNP) in the *DRD2* gene (C957T, rs6277), has been associated with changes in DRD2 availability and DA neurotransmission (37–39), and T-allele homozygosity has been found highly correlated with BED risk and enhanced DA neurotransmission (34). Moreover, the A-allele (also called A1/A1 or A1/A2 genotype) of rs1800497 SNP, in the *ANKK1* gene, has been associated with a reduction of D2/D3 receptors expression and activity (33, 40) and it was significantly more frequent in the obese group than in lean controls (36), contrarily to patients with BED that showed a higher proportion of homozygotes GG (A2-allele) (34). The *DRD4* gene (MIM#126452), localized on chromosome 11p15.5 is highly polymorphic and it is one of the most studied genes not only *in vitro*, but also in animal models (41, 42). Several studies have demonstrated a correlation between EDs and an SNP located in the promoter region of *DRD4* (C521T, rs1800955); notably, the C allele was associated with personality traits related to AN, and in particular, is correlated with perfectionism (42, 43). *DRD4* gene contains four exons and the third one has a polymorphic sequence characterized by a variable number of tandem repeats, known as 48-bp VNTR. Allele frequencies of different repeats differ among several human populations (44, 45). The 4-repeat (4R) is the most common repeat in the human population (more than 60%), followed by 7-repeat (7R) (around 20%) and 2-repeat (2R) alleles. The most interesting allele is 7R that has been shown to reduce the expression of the receptor (46) and its affinity for DA (47). This repeated polymorphism has been strongly associated with overeating, obesity (48–51), drug abuse, and related comorbidities (19). Women carrying at least one copy of 7 or more repeats (>7R) showed an increased body mass index (BMI), with the highest BMI values observed in 7R/7R homozygotes (52). The same genotype (7R/7R) was also associated with a greater risk for AN [odds ratio (OR) = 3.83] (53). Another interesting polymorphism is the 120-base pair (bp) TR, located in the promoter region of *DRD4*. This polymorphism consists of a long (L) and a short (S) allele with opposite effects on the *DRD4* expression level: the L-allele is a negative modulator whereas the S-allele induces higher transcriptional levels of the gene (54). Interestingly, the S-allele has been associated with the AN binging/purging subtype but not with the AN restrictive subtype (42). Moreover, patients with AN carrying the S-allele displayed significantly higher weight and height than patients not having the S-variant (53). Strong evidence suggest also a correlation between *DRD4* and some psychopathological features in patients with BN (55). Different SNPs in the *DRD4* gene may predispose to BN, aggravate its clinical course, or reflect other comorbidities in these patients (49).

In this complex scenario, the aim of this study was to further explore the role of the DA pathway in the genetic susceptibility to the three main EDs in a large, strictly selected cohort of Italian patients affected by AN (*n* = 332), BN (*n* = 122), and BED (*n* = 132), compared to healthy controls (*n* = 172). Six functional polymorphic sites in the *DRD2, ANKK1*, and *DRD4* genes have been analyzed: four SNPs (*DRD2 ANKK1*-rs1800497, *DRD2*-rs6277, *DRD4*-rs936461, and *DRD4*-rs1800955), one TR (*DRD4*-120bp), and one VNTR (*DRD4*-48bp TR). Allelic and genotypic frequencies have been correlated not only with the risk of developing EDs but also with psychopathological and clinical comorbidities often observed in patients.

## Materials and Methods

### Ethics Statement

The clinical sample consisted of 586 Caucasian unrelated patients with ED and 172 healthy controls (CTRs) without a lifetime history of EDs and/or other psychiatric disorders and substance abuse, consecutively recruited in Italy. All participants provided written informed consent prior to inclusion in this project and were treated in accordance with the Declaration of Helsinki. For data protection and confidentiality, all participants were assigned with a unique research identifier alpha-numeric code. The study protocol and the research process were assessed and approved by the Ethics Committee of the Aziende Sanitarie (CEAS) della Regione Umbria, Italy.

### Patients and CTRs Recruitment

EDs subjects were admitted in the residential facility for at least 3 months. They all fulfilled the DSM-V criteria for EDs and were divided into three major groups: AN restrictive subtype (*n* = 332), BN (*n* = 122), and BED (*n* = 132). The clinical diagnosis was verified by psychiatrists with a long expertise in EDs. They used the DSM-V criteria and assessed patients during a face-to-face interview. The evaluation of patient's psychopathological features was performed during the first week of hospitalization in the Department of Eating Disorder, Palazzo Francisci (USL 1 Umbria, Todi, Italy). Exclusion criteria for the admission, determined upon screening, included mental retardation, dementia, schizophrenia, Turner's syndrome, other neurological disorders, and underlying endocrine pathologies.

The study protocol was approved by the Ethics CEAS della Regione Umbria (Italy) and was conducted in accordance with the Declaration of Helsinki and its subsequent revisions. Blood samples of all patients were collected from October 2012 to November 2019 in the Department of Eating Disorder, Palazzo Francisci. After obtaining their consent, specific questionnaires and tests were provided to every patient to define the clinical characteristics (Table 1). All participants were white Italian subjects with a severe ED diagnosis. Gender, age, BMI, and all clinical characteristics of patients with EDs are reported in Table 1. The occurrence of binge eating and self-induced vomiting, have been reported when the episodes were at least seven per week to stress a severe or extreme condition.

**Table 1:** Clinical characteristics of patients with eating disorders (EDs) [anorexia nervosa (AN), bulimia nervosa (BN), and binge eating disorder (BED)] included in this study.

	**AN**	**BN**	**BED**
Sex (% female)	98.2	95.9	88.5
Age years (mean ± SD)	22.48 ± 8.85	26.68 ± 9.7	40.06 ± 19.18
BMI (mean ± SD)	14.3 ± 1.98	18.2 ± 4.62	42.33 ± 10.8
Secondary Amenorrhea (%)	88.5	52.9	20.4
Amenorrhea duration in months (mean ± SD)	29.6 ± 42.8	17.35 ± 18.71	7.9 ± 7.67
Menarche age in years (mean ± SD)	12.59 ± 2.35	12.74 ± 2.39	12.03 ± 1.62
Food restriction (%)	98	93.9	50.7
Water restriction (%)	43.4	25.5	13.6
Fasting	66.9	67.6	25.4
Diet pills (%)	8.8	28.2	23.5
Binge eating ≥7 episodes/week (%)	–	96	80.3
Vomit ≥7 episodes/week (%)	–	91	3.2
Alcohol abuse (%)	12.1	10	5.4
Laxative abuse (%)	28.6	39.6	9
Diuretics abuse (%)	7.2	16	7.6
Drug abuse (%)	7	16.3	10.4
Excessive physical activity (%)	75.5	60	20.3

In addition, 172 CTRs from the same geographical area as the patients, were recruited. Interviews and physical examinations were carried out to ensure that they had never been diagnosed as having any kind of EDs and/or psychiatric disorders and to ensure that the BMI was in the range of normality during all their life (18.5–24.9 kg/m^2^).

### Genomic DNA Extraction

Genomic DNA was isolated from 300 μl of whole anticoagulated peripheral blood samples using a commercially gDNA Mini Kit (Geneaid) according to the manufacturer's instructions. The gDNA was quantified using the NanoDrop system and 100 ng of each sample was run in 0.8% agarose gel electrophoresis in 1X TBE buffer at 90 V for 45 min to verify the integrity of gDNA.

### Genotyping of DRD2 and DRD4 SNPs

Genotyping of DRD2/ANKK1-rs1800497, DRD2-rs6277, DRD4-rs936461, and DRD4-rs1800955 has been performed using the following TaqMan SNP Genotyping Assays (Thermo Fisher Scientific, Waltham, MA, USA): C___7486676_10; C__11339240_10; C___7470693_30; C___7470700_30. Ten ng of the template gDNA was used for the assays. All the samples were analyzed with 7500 Real-Time PCR (Applied Biosystem) apparatus using the recommended cycling conditions: denaturation phase (95°C for 15 min) followed by annealing and extension 50 cycles (60°C for 1 h and 30 min), as previously described (56). Each run was performed including positive controls (wildtype, heterozygous, and homozygous variant genotypes).

### Genotyping of DRD4-120bp TR and DRD4-48bp VNTR

The DRD4-120bp TR polymorphism was assayed according to methods described by Seaman et al. (45). Primer sequences used are: sense 5′-GTTGTCTGTCTTTTCTCATTGTTTCCATTG−3′ and antisense 5′-GAAGGAGCAGGCACCGTGAGC-3′. The amplification products were electrophoresed on a 2% agarose gel and visualized by ethidium bromide staining. The PCR reaction yields distinct bands at 429 bp (120-bp S, short allele) and 549 bp (120-bp L, long allele). The exon 3 DRD4-48bp VNTR polymorphism was assayed as described by Mitsuyasu et al. (57), with slight modifications. PCR amplification was performed in a volume of 30 μl containing 50 ng genomic DNA; 1 μM of each primer; 100 μM each of dATP, dTTP, dCTP, and 7-deaza-2′-deoxyguanosine 5′-triphosphate; 1.5 mM MgCl2; 10% DMSO; and 1 unit of GoTaq Hot Start Polymerase (Promega Corporation, Madison, Wisconsin, USA). The primer sequences used were: sense 5′-AGGTGGCACGTCGCGCCAAGCTGCA-3′; antisense, 5′-TCTGCGGTGGAGTCTGGGGTGGGAG-3′. This analysis discriminates variant alleles containing one (1R) to ten (10R) repeats, which have been secondly grouped according to the presence or not of the 7R or more repeats alleles. DNA was denatured at 99°C for 2 min prior to the addition of other components. The master mix was assembled and incubated with the samples, then, 40 cycles (95°C for 20 s, 56°C for 30 s, and 72°C for 50 s), with a final step at 72°C for 7 min were performed in a Veriti 96-well thermal cycler (Thermo Fisher Scientific, Massachusetts, USA). The PCR products were electrophoresed on a 3% agarose gel and visualized by ethidium bromide staining. Random samples were sequenced for length confirmation.

### Statistical Analysis

Genotypes frequencies were tested for the Hardy—Weinberg equilibrium (HWE). Differences in genotypes and alleles frequencies between cases and controls were evaluated by Pearson's chi-squared test. A *p* ≤ 0.05 was considered significant. A correlation analysis between the genotypes and the clinical/phenotypes was performed first in each ED group and after in the whole cohort of patients with ED. Since we have analyzed eleven clinical variables (the behavioral phenotypes), we have used a Bonferroni correction to determine the significance threshold (*P* < 0.005). ORs and 95% CIs were calculated. The ANOVA test was used to compare BMI values among the different genotypic classes. All statistical analyses were performed by the SPSS program, version 25 (IBM Corporation, Armonk, New York, USA). In addition, haplotypes for significantly associated polymorphisms (DRD2-rs6277, DRD4-rs936461, 48-bp VNTR, and 120-bp TR) were inferred using the Haploview version 4.2 program. Differences in haplotype distribution between cases and controls were evaluated by the chi-squared test.

## Results

### Demographic and Behavioral Characteristics of the Study Cohort

Our analysis included 586 patients affected by EDs: 332 patients with AN restrictive subtype, 122 patients with BN and 132 patients with BED, and 172 patients with CTRs. Descriptive analyses of the demographic and behavioral data of the participants are given in Table 1. All the subjects during recruitment have filled out a questionnaire with different queries about: sex, age, BMI, secondary amenorrhea, amenorrhea duration, menarche age, food restriction, water restriction, fasting, diet pills, binge eating, vomit, alcohol abuse, laxative abuse, diuretics abuse, drug abuse, and excessive physical activity. “Yes” or “No” was the first possible range of responses. If “Yes,” they might indicate total months for secondary amenorrhea duration and for the other answers (food restriction, water restriction, fasting, diet pills, binge eating, vomit, alcohol abuse, laxative abuse, diuretics abuse, and drug abuse) might specify the number of episodes per week.

The age at onset was 22.48 ± 8.85 in AN, 26.68 ± 9.7 in BN, and 40.06 ± 19.18 in BED. The mean age at recruitment for CTRs was 33.16 ± 10.87. Women were prevalent in patients with EDs and in CTRs with the following frequencies: 98.2% in AN, 95.9% in BN, 88.5% in BED, and 84.5% in CTRs. The BMI values (kg/m^2^) at hospitalization were 14.3 ± 1.98 for AN, 18.2 ± 4.62 for BN, and 42.33 ± 10.8 for BED. Moreover, 76.2 % of AN had a BMI <16 kg/m^2^. This testifies that 3 out 4 patients with AN had a severe or extreme severity, based on BMI value (DMS-V). At the same time also some patients with BN showed a BMI under 18.5 kg/m^2^ and this is possible due to a possible diagnostic crossover from AN purging subtype and BN during the lifetime history of the disease. The CTRs had a normal BMI (21.26 ± 2). The secondary amenorrhea was prevalent in AN (88.5%), followed by BN (52.9%) and BED (20.4%). The primary amenorrhea was not required as it does not increase diagnostic specificity and moreover, it has been removed as a diagnostic criterion in DMS-V. Food restriction was a behavioral characteristic present in almost all AN (98%), in a large group of BN (93.9%) and in half of the BED group (50.7%). When we referred to dietary restriction behavior, we want to mean a qualitative and quantitative behavior: eating fewer meals per day, higher frequency of fasting, consuming small meals, and only low-calorie foods. In the survey, the criterion for food restriction to standardize all answers is: eating <1,000 kcal/die and patients may answer Yes or No. Water restriction was a condition almost present in extreme patients (43.4% of AN, 25.5% of BN, and 13.6% of BED). Patients, in particular affected by AN, decide to reduce water intake for the incessant worry over gaining weight. But sometimes, all the biological functions are impaired and patients lose the sense of thirst. In any case, the water restriction is indicated in the questionnaire as drink <0.5 L/die. In the survey, fasting common in AN and BN (around 67%) is defined as skipping at least one meal/day. Binge eating was not present in patients with AN, but were common traits for BN and BED with an incidence of 7 or more episodes/week in 96% of BN, and in 80.3% of BED, respectively. Inappropriate compensatory behavior (self-induced vomiting) was absent in AN and present in 91% of BN and 3.2% of BED. Seven episodes/week threshold for binge eating and purging episodes have been chosen because we wanted to include only patients with a severe or extreme grade of disease. In particular, in BN when the inappropriate compensatory behaviors are ≥ 7/week the clinical picture is very severe: electrolyte decompensation, such as sodium, and sometimes gastrointestinal problems.

Alcohol, diuretics, and drug abuse were unusual, in general present <10% in all patients. The laxative abuse was the most common with an incidence of 28.6% in AN, 39.6% in BN, and 9% in BED. The excessive physical activity is linked to a large portion of patients, in particular, AN (75.5%) and BN (60%), less present in BED (20.3%).

### Genotypes and Alleles Frequencies of Polymorphisms in DRD2 and DRD4 Genes

In this study, we have investigated 4 common SNPs (*DRD2/ANKK1*-rs1800497; *DRD2*-rs6277; *DRD4*-rs936461; *DRD4*-rs1800955), one TR (*DRD4*-120bp TR), and one VNTR (*DRD4*-48 bp VNTR) and their possible association with EDs in a large cohort of Italian patients. We have not observed deviations from HWE for all the genetic variants analyzed in the CTRs. The distribution of the different genotypes and alleles frequencies in patients with AN, BN, and BED and in CTRs is shown in Table 2.

**Table 2:** *ANKK1, DRD2*, and *DRD4* genotypes and alleles distribution in ED patients (AN, BN, and BED) and healthy controls (CTRs): casecontrol association analysis.

	**Genotypes**			***N***	*P* ^*^	*P* ^**^	OR^**^(95%CI)	**Alleles**	*P* ^***^	OR^***^(95% CI)	
**ANKK1 rs1800497**	**GG**	**GA**	**AA**					**G**	**A**		
AN	221 (66.6%)	102 (30.7%)	9 (2.7%)	332	0.17	0.16	1.34 (0.89–2)	544 (72%)	120 (18%)	0.35	1.18 (0.83–1.66)
BN	90 (73.8%)	31 (25.4%)	1 (0.8%)	122	0.23	0.84	0.95 (0.56–1.6)	211 (86.5%)	33 (13.5)	0.47	0.84 (0.53–1.34)
BED	97 (73.5%)	34 (25.8%)	1 (0.8%)	132	0.19	0.88	0.96 (0.58–1.6)	228 (86.4%)	36 (13.6%)	0.48	0.85 (0.54–1.34)
CTRs	125 (72.7%)	40 (23.3%)	7 (10.1%)	172				290 (84.3)	54 (15.7%)		
**DRD2 rs6277**	**AA**	**AG**	**GG**					**A**	**G**		
AN	108 (32.5%)	174 (52.4%)	50 (15.1%)	332	**0.008**	**0.002**	1.80 (1.24–2.63)	390 (58.7%)	274 (41.3%)	**0.011**	1.42 (1.08–1.86)
BN	31 (25.4%)	68 (55.7%)	23 (18.9%)	122	**0.001**	**0.0003**	2.55 (1.54–4.23)	130 (53.3%)	114 (46.7%)	**0.0009**	1.77 (1.26–2.48)
BED	49 (37.1%)	60 (45.5%)	23 (17.4%)	132	0.22	0.1	1.47 (0.93–2.34)	158 (59.8%)	106 (40.2%)	0.14	1.31 (0.92–1.86)
CTRs	80 (44.5%)	70 (40.7%)	22 (12.8%)	172				230 (66.9%)	114 (33.1%)		
**DRD4 rs936461**	**GG**	**GA**	**AA**					**G**	**A**		
AN	107 (32.2%)	165 (49.7%)	60 (18.1%)	332	0.66	0.37	0.83 (0.56–1.24)	379 (57.1%)	285 (42.9%)	0.4	0.89 (0.69–1.16)
BN	53 (43.4%)	52 (42.6%)	17 (13.9%)	122	**0.025**	**0.007**	0.51 (0.32–0.84)	158 (64.8%)	86 (35.2%)	**0.011**	0.65 (0.46–0.91)
BED	56 (42.4%)	52 (39.4%)	24(18.2%)	132	**0.031**	**0.01**	0.54 (0.33–0.87)	164 (62.1%)	100 (37.9%)	**0.05**	0.73 (0.52–1)
CTRs	49 (28.3%)	90 (52%)	34 (19.7%)	172				188 (54.3%)	158 (45.7%)		
**DRD4 rs1800955**	**TT**	**TC**	**CC**					**T**	**C**		
AN	91 (27.2%)	168 (50.8%)	73 (22.1%)	332	0.65	0.60	0.89 (0.98–1.36)	350 (52.7%)	314 (47.3%)	0.37	0.89 (0.68–1.15)
BN	39 (32%)	60 (49.2%)	23 (18.9%)	122	0.26	0.19	0.71 (0.42–1.19)	138 (56.6%)	106 (43.4%)	0.1	0.76 (0.55–1.06)
BED	24 (18.2%)	77 (58.3%)	31 (23.5%)	132	0.24	0.16	1.5 (0.86–2.63)	125 (47.4%)	139 (52.6%)	0.57	1.1 (0.8–1.52)
CTRs	43(25%)	85(49.4%)	44(25.6%)	172				171 (49.7%)	173 (50.3%)		
**VNTR 120bp**	**L**	**LS**	**S**					**L**	**S**		
AN	162 (50.3%)	142 (44.1%)	18 (5.6%)	322	0.12	0.17	0.77 (0.53–1.12)	466 (72.4%)	178 (27.6%)	0.07	0.77 (0.58–1.02)
BN	65 (54.6%)	48 (40.3%)	6 (5%)	119	0.11	0.07	0.65 (0.40–1.03)	178 (74.8%)	60 (25.2%)	**0.04**	0.68 (0.47–0.98)
BED	30 (22.7%)	98 (74.2%)	4 (3%)	132	**<0.001**	**0.0001**	2.65 (1.59–4.4)	158 (60.4%)	106 (39.6%)	0.08	1.35 (0.97–1.89)
CTRs	74 (43.8%)	78 (46.2%)	17 (10.1%)	169				226 (66.9%)	112 (33.1%)		

* *Comparison among the three genotypic classes*.

** *Comparison among heterozygotes and variant homozygotes (combined together) vs. wildtype homozygotes*.

*** *Comparison among alleles*.

*P-values in bold indicate significant associations.*

Among the analyzed polymorphisms, the case-control association analysis revealed significant associations for the *DRD2*-rs6277 and *DRD4*-rs936461 SNPs. The variant allele (C) of rs6277 SNP in *DRD2* gene seems to be a risk allele; it was significantly more present in patients with AN and then in CTRs, with an OR = 1.42 (95% CI 1.08–1.86; *p* = 0.011) and OR = 1.77 (95% CI 1.26–2.48; *p* = 0.0009), respectively. Whereas, the minor allele (A) of *DRD4*-rs936461, was associated with a lower risk of developing BN and BED, with an OR= 0.65 (95% CI 0.46–0.91; *p* = 0.011) and OR = 0.73 (95% CI 0.52–1; *p* = 0.05), respectively. A significant association was found between the *DRD4*-120bp TR polymorphism and the BED susceptibility. Indeed, the homozygous and heterozygous genotypes (SS plus LS genotypes) were significantly more present in BED than in CTRs (OR = 2.65; 95% CI 1.59–4.4; *p* = 0.0001). Genotyping of *DRD4*-48bp VNTR polymorphism identified 9 different alleles (Supplementary Table 1): from one (1R) to ten (10R) repeats. Among them, only the 7R or more (≥ 7R) alleles are associated with a higher risk of developing EDs. The distributions of subjects carrying no ≥ 7R allele, one ≥ 7R allele, or two ≥ 7R alleles, are shown in Table 3.

**Table 3:** Analysis of *DRD4*-48 bp VNTR: comparison of the frequency of 7 or more repeats (≥7 R) alleles in cases (AN, BN, and BED) and healthy controls (CTRs).

	**Presence of 7R (or more repeats) alleles**			***P*_3cat_**	***P*_2cat_**	**OR (95% CI)**
	**Number of subjects with no allele ≥7R**	**Number of subjects with one allele ≥7R**	**Number of subjects with two alleles ≥7R**			
AN	244 (79%)	56 (18.1%)	9 (2.9 %)	0.028	0.008	2.09 (1.2–3.62)
BN	81 (75%)	25 (23.1%)	2 (1.9%)	0.008	0.003	2.6 (1.67–4.99)
BED	82 (73.9%)	26 (23.4%)	3 (2.7%)	0.005	0.001	2.77 (1.47–5.25)
CTRs	149 (88.7%)	16 (9.5%)	3 (1.8%)			

*P_2cat_, The analysis has been performed combining together subjects carrying 1 copy or 2 copies of 7R (or more) alleles in comparison with subjects carrying no 7R (or more) alleles (including therefore 1R, 2R, 3R, 4R, 5R, and 6R)*.

The presence of ≥7R allele seems to be associated with a higher risk to develop each type of EDs (AN: OR = 2.09; 95% CI 1.2–3.62; *p* = 0.008; BN: OR = 2.6; 95% CI 1.67–4.99; *p* = 0.003; BED: OR = 2.77; 95% CI 1.47–5.25; *p* = 0.001). We also inferred the haplotypes among the *DRD2*-rs6277, *DRD4*-rs936461, *DRD4*-48bp VNTR, and *DRD4*-120bp TR, but the comparison of haplotypes combinations between patients with ED and CTRs did not improve the statistical significance obtained from single SNPs association analysis (see Supplementary Figure 1).

**Figure 1:**
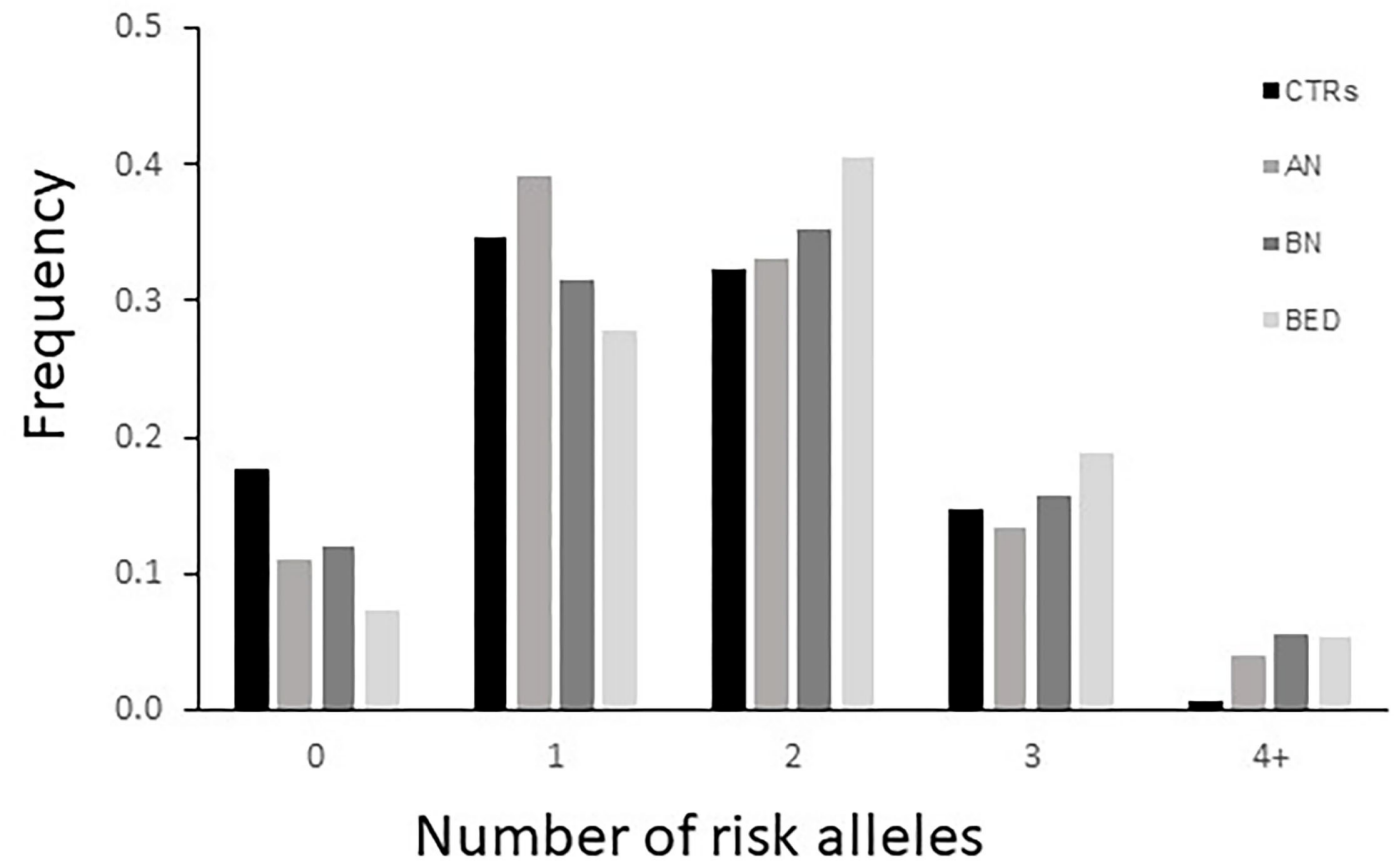
Distribution of *DRD2* and *DRD4* risk alleles risk in cases [patients with anorexia nervosa (AN), bulimia nervosa (BN), and binge eating disorder (BED)] and healthy controls (CTRs). The variant allele of *DRD2*-rs6277, ≥7 repeats of the *DRD4*- 48bp variable number tandem repeat (VNTR), and S allele of *DRD4*-120bp TR were included as risk alleles.

Finally, we have performed a counting of the risk alleles comparing their distribution between cases and controls (Figure 1). In this analysis, the following has been considered as risk alleles: C variant allele of *DRD2*-rs6277, 48bp VNTR ≥7 repeats, and 120 bp L allele in *DRD4* gene. As expected, the class with four or more risk alleles was significantly more present in cases than in CTRs, showing a significantly higher risk to develop EDs for subjects carrying a higher load of such alleles [*p* = 0.003, OR = 6.74 (0.87–52.34) in AN; *p* = 0.017 and OR = 9.59 (1.14–80.8) in BN; *p* = 0.02 and OR = 9.31 (1.11–78.47) in BED].

### Correlation Analyses Between DRD2/DRD4 Genotypes and Behavioral/Clinical Phenotypes of Patients With ED

Our cohort of patients consists of three EDs subtypes (AN, BN, and BED) typically showing a wide variety of behavioral and clinical associated comorbidities. We, therefore, decided to carry out a genetic correlation analysis between *DRD2* and *DRD4* genetic polymorphisms and the following behavioral phenotypes: diuretics, diets pills, laxative and drug abuses, food restriction, water restriction, fasting, alcohol abuse, excessive physical activity in all three groups, while vomit and binge eating reward with a frequency of ≥7 times/week observable in patients with BN ad BED only. Significant results are shown in Table 4.

**Table 4:** Significant findings of the genotype–phenotype correlation analysis.

**AN**	**Phenotype**	***p***	**OR (95% CI)**
DRD4 rs1800955 (TT vs. TC + CC)	Diuretics abuse	0.003	3.85 (1.52–10)
**BN**			
DRD4 VNTR 120bp (LS + S vs. L)	Diets pills	0.002	4.17 (1.61–10.76)
**BED**			
ANKK1 rs1800497 (GG vs. GA + AA)	Binge eating	0.001	7.69 (2.08–29.4)
**All cases**			
DRD4 rs1800955 (TT vs. TC + CC)	Diuretics abuse	0.001	2.86 (1.47–5.55)
DRD4 VNTR 120bp (LS + S vs. L)	Diets pills	<0.001	2.55 (1.45–4.48)
	Drugs abuse	0.001	3.19 (1.52–6.71)

A significant association was found between the TT genotype of *DRD4*-rs1800955 and diuretics abuse in patients with AN (OR = 3.85; 95% CI 1.52–10; *p* = 0.003). In patients with BN, those carrying the *DRD4*-120bp TR S-allele showed a higher predisposition to abuse of diet pills (OR = 4.17; 95% CI 1.61–10.76; *p* = 0.002). Patients with BED homozygous for the G-allele of *ANKK1*-rs1800497 exhibited a significant association with binge eating episodes (OR = 7.69; 95% CI 2.08–29.4; *p* = 0.001). Correlation analysis in the whole patients with EDs cohort further confirms a significant association between *DRD4*-rs1800955 (TT genotype) and diuretics abuse (OR = 2.86; 95% CI 1.47–5.55; *p* = 0.001). Moreover, patients with EDs with at least one copy of the *DRD4*-120bp TR short-allele showed a higher susceptibility to abuse of diets pills (OR = 2.55; 95% CI 1.45–4.48; *p* < 0.001) and drugs (OR = 3.19; 95% CI 1.52–6.71; *p* = 0.001).

**Figure 2:**
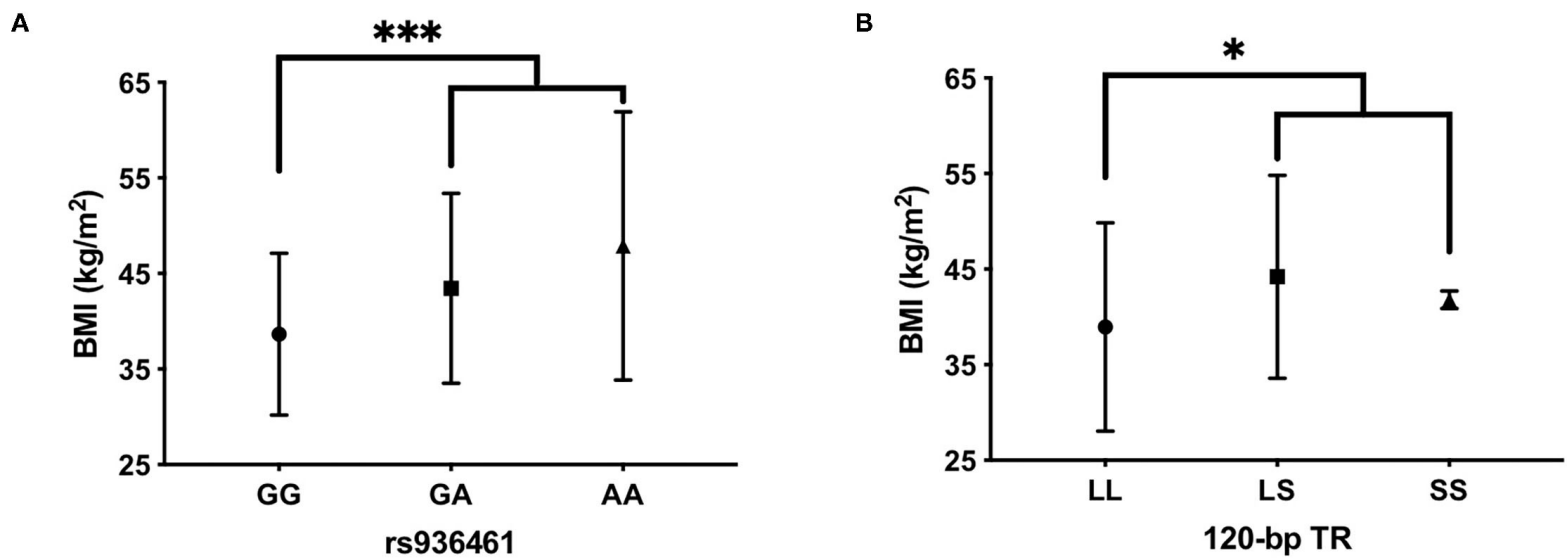
Evaluation of body mass index (BMI) among carriers of *DRD4*- rs936461 GG, GA, and AA genotypes **(A)** and *DRD4*-120bp TR LL, LS, and SS genotypes **(B)** in BED patients. Statistical comparisons were performed by one-way ANOVA with *post-hoc* correction by the Tukey method. ***p < 0.001 vs. carriers of rs936461A-allele and *p < 0.05 vs. carriers of DRD4-120bp TR short-allele. All values are mean ± SD.

Our correlation analysis also included the BMI expressed in kg/m^2^. BMI was under the normal cut-off value of 18.5 in AN (mean = 14.3 ± 1.98), almost in the range with few exceptions in BN (mean = 18.2 ± 4.62) and always above 30 (the limit of overweight) in patients with BED (mean = 42.33 ± 10). ANOVA analysis was used to investigate the association between *DRD2* and *DRD4* polymorphisms and BMI values in each ED group. Interestingly, patients with BED carrying the *DRD4*-rs936461 A allele showed a higher BMI value compared with GG homozygotes (*p* < 0.0001, Figure 2A). In addition, the patients with BED carrying the *DRD4*-120bp TR short-allele (LS plus SS genotypes) showed statistically significant higher BMI than homozygotes for the 120 bp TR long-allele (LL genotype) (*p* = 0.04, Figure 2B). There were no significant genetic correlations of *DRD2* and *DRD4* with BMI values in AN and BN groups.

## Discussion

The increasing number of publications reporting genetic association studies in patients with ED has produced both important true association results and negative or controversial results. Most of the reported discrepancies can be justified by the heterogeneity of the clinical phenotypes and by the different ethnicities of the individuals analyzed. One major strength of our work is the selection of three homogeneous large cohorts of patients with EDs exclusively recruited in Italy compared to strictly selected age and sex-matched controls. These sample cohorts have been already studied by our group (58) demonstrating a positive association between EDs susceptibility and two candidate genes: *5-HT2AR* and *BDNF* (56). In this report, we further broaden these findings with the analysis of the *DRD2* and *DRD4* genes, encoding for DRDs already associated with AN and BED susceptibility (34, 52, 59). We found that the C allele of rs6277 in the *DRD2* gene represents a risk for developing AN and BN, but not BED, in accordance with previous results (34, 59). Indeed, the C allele of rs6277 (C957T) has been associated with AN susceptibility (59) whereas a significantly higher frequency of the TT genotypes was found in patients with BED (34). The rs6277 polymorphism can affect the folding of *DRD2* mRNA *in vitro* with functional effects on the stability and the translation efficiency of *DRD2* transcript (37). The presence of rs6277 T-allele has been associated with a relevant decrease in the protein synthesis level indicating a reduction in *DRD2* mRNA translation efficiency (37). Furthermore, human studies have demonstrated that the C allele is associated with both low striatal DRD2 availability (38) and with high extra-striatal DRD2 density throughout the cortex and the thalamus (39). These observations indicate that the rs6277 C allele could regulate the DRD2 availability in different brain regions thus explaining the clinical features linked to the dopaminergic system dysregulation observed in patients with ED (39, 60). For the first time, we observed a strong association between rs936461 SNP (A809G) in the promoter region of *DRD4* gene and two specific EDs subgroups: BN and BED. Patients carrying the minor rs936461 A-allele are less prone to develop both BN and BED. Moreover, we also found an interesting relationship of this SNP with BMI in BED, with the highest BMI values in AA homozygotes. *DRD4* promoter region SNPs have been previously investigated in a trios study, including 202 AN women, mainly from Israel (divided in binging/purging and restrictive subtypes) showing biased non-transmission of rs936461 A-allele and only a partial transmission (no significance was found in AN restrictive subtype) of rs1800955 C-allele (42). In our case-control study, we did not confirm the association of rs1800955 C-allele with AN. However, the different populations analyzed and the different approaches used for the association study (trios vs. case-control study) make the above-reported study hardly comparable with the present work.

A significant association between the *DRD4*-120bp TR polymorphism and BED susceptibility was found. Indeed, SS and SL genotypes were significantly more present in BED than in CTRs. The location of the polymorphism in the 5' regulatory region of the *DRD4* gene within potential transcription factor binding sites suggests that different alleles might confer differential transcriptional activity of the gene. Indeed, the longer L allele has lower transcriptional activity than the shorter S allele (54). The 120-bp TR polymorphism has been previously reported to influence cue-elicited craving for food, with *DRD4* L allele carriers showing significantly decreased levels of craving on subsequent eating trials compared to *DRD4* S allele carriers (26). Reactivity to food cues is associated with binge eating and BMI (61). Accordingly, we found a higher BMI in patients with BED carrying SS or SL genotypes compared to LL homozygotes, suggesting that this polymorphism in the *DRD4* gene could influence cue-elicited craving for food. We also found a strong association of ≥7R alleles of the *DRD4*-48bp VNTR with all three subgroups of EDs, with the highest significance in BED subjects. Our data are in line with previous reports (49, 50, 60) and confirm that genetic variations in the DA pathways are able to modify the overall risk for EDs. The ≥7R alleles have been associated with binge eating behavior in women with seasonal affective disorders (50) and influence body weight regulation in women with BN (48). Compulsive food intake has been associated with low brain DA release and activity in particular in Ventral Tegmental Area (VTA) (62–64). In obese rodents, this condition has been linked to compulsive food intake (65). Preclinical and clinical studies in humans have provided evidence of decreases in DA signaling in VTA that was related to a dramatic increase in the consumption of high-fat foods (63, 64). In this context, previous studies have indicated that the presence of 7R allele of *DRD4*-48bp VNTR has been associated with a reduced expression and activity of the receptor (46, 47). The 7R variant encodes for a receptor with a lower intracellular response to DA, and 7R carriers might be more tempted to overconsume drugs or palatable food thus enhancing DA levels in the brain. Several conflicting results emerge from this study, one is the lack of association between the ANKK1-rs1800497 and EDs. The A-allele (sometimes indicated A1) has been often associated with obesity, abnormal feeding, and craving behavior, such as abuse of alcohol and cocaine (27, 36, 66). Obesity and BED have common characteristics but they are unrelated disorders that need to be distinguished also for a personalized therapeutic approach. In fact, overeating in patients with BED is a result of brain reward cascade dysfunction, usually linked with hypodopaminergic function and ANKK1 may play an important role in modifying dopaminergic signaling (67). Indeed, Davis and co-workers found a prevalence of G/G (A2/A2) genotype in patients with BED (34) and Palacios et al. indicated that individuals with binge eating phenotype presented the G allele more frequently, in comparison with patients with obesity and no BED phenotype (68). Among 132 patients with BED analyzed, we only found a significant association of the G allele within the group that declared to resort to binge eating episodes ≥7 times/week. We, therefore, hypothesize that the rs1800497 SNP could alter the reward pathways leading to a wide spectrum of addictive, compulsive, and impulsive behaviors typical of binge eating episodes observed in our cohort of patients with BED.

Data on the influence of DA genes polymorphisms on the psychopathological features displayed by patients with EDs are still lacking, especially in comparison with the abundance of the paper published on serotonergic polymorphisms. Emerging evidence suggest that the neural mechanisms implicated in the appetitive motivation for drugs and those involved in appetitive motivation for the food show the same core features (69, 70). This supports the “food addiction” hypothesis according to which, both drugs of abuse and food consumption activate the same brain pathway where the dopaminergic signaling is the principal neurotransmission system involved in reward and motivation (27, 69). Indeed, pathologically obese individuals showed a reduced level of striatal DRD2 similarly to drug-addicted subjects, likely reflecting their decreased maturation and surface expression. Moreover, DRD2 was found to be inversely correlated with the BMI in patients with obesity (71, 72). It is therefore possible to that the decrease in DRD2 predispose to search for reinforces, either drug in drug-addicted subjects or food in patients with obesity, to temporarily compensate for a decreased sensitivity of DA rereward circuits. Recently, *DRD4* has been suggested to be an attractive target for the treatment of neuropsychiatric diseases (73–77), including EDs, but more information is needed. For the first time, we report a correlation between the *DRD4*-rs1800955 SNP and diuretics abuse in the three cohorts of patients with ED, not only in patients with AN, as previously reported. This SNP is located within the *DRD4* promoter region and Okuyama et al. showed a reduced transcriptional efficiency of the T allele compared to the −521 C variant in human retinoblastoma cell lines, suggesting a role in dopaminergic neurotransmission (78). It is therefore possible to argue that the positive association between TT homozygous subjects and diuretic abuse in our ED cohort could be explained by a behavioral phenotype linked to a severe DA neurotransmission dysfunction. Interestingly, the *DRD4*-120bp TR polymorphism, already linked with BED and with higher BMI in our analysis, was also associated with diets pills and drugs abuse predisposition. Among patients with BN, those carrying the S allele (LS plus SS genotypes) showed a higher risk to use diet pills. This tendency is likewise present in the whole cohort of patients with EDs that show a higher predisposition not only for diet pills but also to drugs abuse. As previously described in the text, this link could be ascribed to the enhanced transcriptional activity of the short allele compared with the long one (54). Patients carrying SS or SL genotype are more prone to drugs abuse suggesting a link between the *DRD4*-120bp TR polymorphism and drug-related cues for obtaining a reward. This study might also provide some useful issues regarding the possibility to develop novel therapeutic compounds for EDs and associated phenotypes. The treatment of these disorders presents several obstacles including patients' compliance with treatment and the limited set of treatments options. Like other psychiatric disorders, interdisciplinary approaches, including cognitive-behavioral therapy (CBT), as well as intrapersonal therapy (IPT), are often combined with pharmacotherapy (79). Pharmacotherapy has the potential to improve EDs pathology outcomes and increase long-term compliance with effective long-term treatment, which is a significant challenge not only for patients, but also for health care providers. Unfortunately, pharmacotherapy options available for EDs are limited and rather rely on the “repurposing” of existing FDA-approved medications for other psychiatric illnesses to include EDs treatment. To date, only BN and BED have benefited from repurposing two existing FDA-approved medications, fluoxetine, and lisdexamfetamine, respectively (80, 81). These treatments are overall safe with some treatment-emergent adverse effects that include insomnia, headache, and an increase in heart rate and blood pressure associated with a longer length of therapy. This “repurposing” approach could be potentially beneficial for finding a pharmacotherapy also for AN and study results for olanzapine, a second-generation antipsychotic, are promising (82). However, even with prolonged clinical treatment aimed at normalizing feeding patterns and attitudes toward food, the remission rates for EDs are still <50%. This underlines the urgent need to develop better research-based treatment options designed at normalizing eating patterns and mitigating factors involved in the susceptibility to EDs. In this context, our results provide further evidence on the importance of the *DRD4* gene as a predisposing factor to develop EDs, making this gene a promising target for the treatment of EDs and more in general of substance use disorders. Indeed, DRD4 is widely expressed in the prefrontal cortex and hypothalamus where it might modulate food intake and eating behavior. The development of selective and potent DRD4 agonists and antagonists might represent a new pharmacological approach for the management of these diseases and have been evaluated in animal studies with unclear results. Experiments on rat models indicate that the use of DRD4 agonists directed injected into the paraventricular nucleus of the hypothalamus, induced hyperphagia whereas the administration of a DRD4 antagonist completely prevented this effect, although it was inactive on food intake when administered alone (83). Therefore, more comprehensive translational research approaches are needed to clarify the influence of DRD4 activation or blockade in food intake and eating behavioral phenotypes.

## Conclusion

In summary, we have shown that variations in genes participating in the DA pathways are able to modify the risk for AN, BN, and BED. Moreover, rs1800955 and 120-bp TR polymorphism of the *DRD4* gene could contribute to some of the psycho-pathological features observed in patients with EDs, in particular the predisposition to diuretic, diet pills, and drug abuse.

DRD4 agonists and antagonists have been indicated as a useful approach in the treatment of drug addiction and other addictive behaviors with unclear results (84). Further functional studies are warranted to establish possible implications of our data in promoting the discovery of new pharmacological compounds for the EDs treatment and their comorbidities. In this scenario, *DRD2* and *DRD4* genes are really attractive and promising targets, in particular for the BED subgroup (85). Olanzapine, a DRD2 antagonist, was already used in AN treatment and other DRDs agonists are under consideration as potential drugs (86).

## Data Availability Statement

The original contributions presented in the study are included in the article/Supplementary Material, further inquiries can be directed to the corresponding author/s.

## Ethics Statement

The studies involving human participants were reviewed and approved by Institutional Review Board Ethics Committee of Aziende Sanitarie (CEAS) della Regione Umbria Prot. No. 29616/12/AV. Written informed consent to participate in this study was provided by the participants' legal guardian/next of kin.

## Author Contributions

MC and TB conceptualized the study. MC, SF, FC, and EG contributed to investigation. MC and SF validated the study. EG contributed to formal analysis. CC contributed to statistical analysis. MC, SF, CC, and MB interpretated the data. MC, SF, CC, and AB contributed to original draft preparation. TB and LD contributed to supervision. MB contributed to project administration. MC, SF, and AB contributed to writing, reviewing, and editing. All authors contributed to the article and approved the submitted version.

## Conflict of Interest

The authors declare that the research was conducted in the absence of any commercial or financial relationships that could be construed as a potential conflict of interest.

## Publisher's Note

All claims expressed in this article are solely those of the authors and do not necessarily represent those of their affiliated organizations, or those of the publisher, the editors and the reviewers. Any product that may be evaluated in this article, or claim that may be made by its manufacturer, is not guaranteed or endorsed by the publisher.
