# Supplementary material for: Association Between DRD2 and DRD4 Polymorphisms and Eating Disorders in an Italian Population
Source: Front Nutr. 2022 Mar 14;9:838177. doi: 10.3389/fnut.2022.838177 (PMC8964431; doi:10.3389/fnut.2022.838177)
Supplement: Supplementary file 1 [file Table_1.DOCX]

|  | 1R | 2R | 3R | 4R | 5R | 6R | 7R | 8R | 10R | Total number alleles |
| --- | --- | --- | --- | --- | --- | --- | --- | --- | --- | --- |
| AN | 0.002 | 0.092 | 0.026 | 0.697 | 0.010 | 0.053 | 0.104 | 0.015 | 0.002 | 618 |
| BN | 0.000 | 0.083 | 0.037 | 0.690 | 0.005 | 0.051 | 0.093 | 0.042 | 0.000 | 216 |
| BED | 0.005 | 0.063 | 0.041 | 0.721 | 0.005 | 0.023 | 0.108 | 0.032 | 0.005 | 222 |
| CTRL | 0.000 | 0.113 | 0.033 | 0.699 | 0.009 | 0.077 | 0.065 | 0.003 | 0.000 | 336 |

Table S1. DRD4 VNTR 48 bp: Frequency of repeats alleles in the different subgroups of cases and in controls
