# Supplementary figures and images for: Association Between DRD2 and DRD4 Polymorphisms and Eating Disorders in an Italian Population
Source: Front Nutr. 2022 Mar 14;9:838177. doi: 10.3389/fnut.2022.838177 (PMC8964431; doi:10.3389/fnut.2022.838177)

Haplotypes distribution

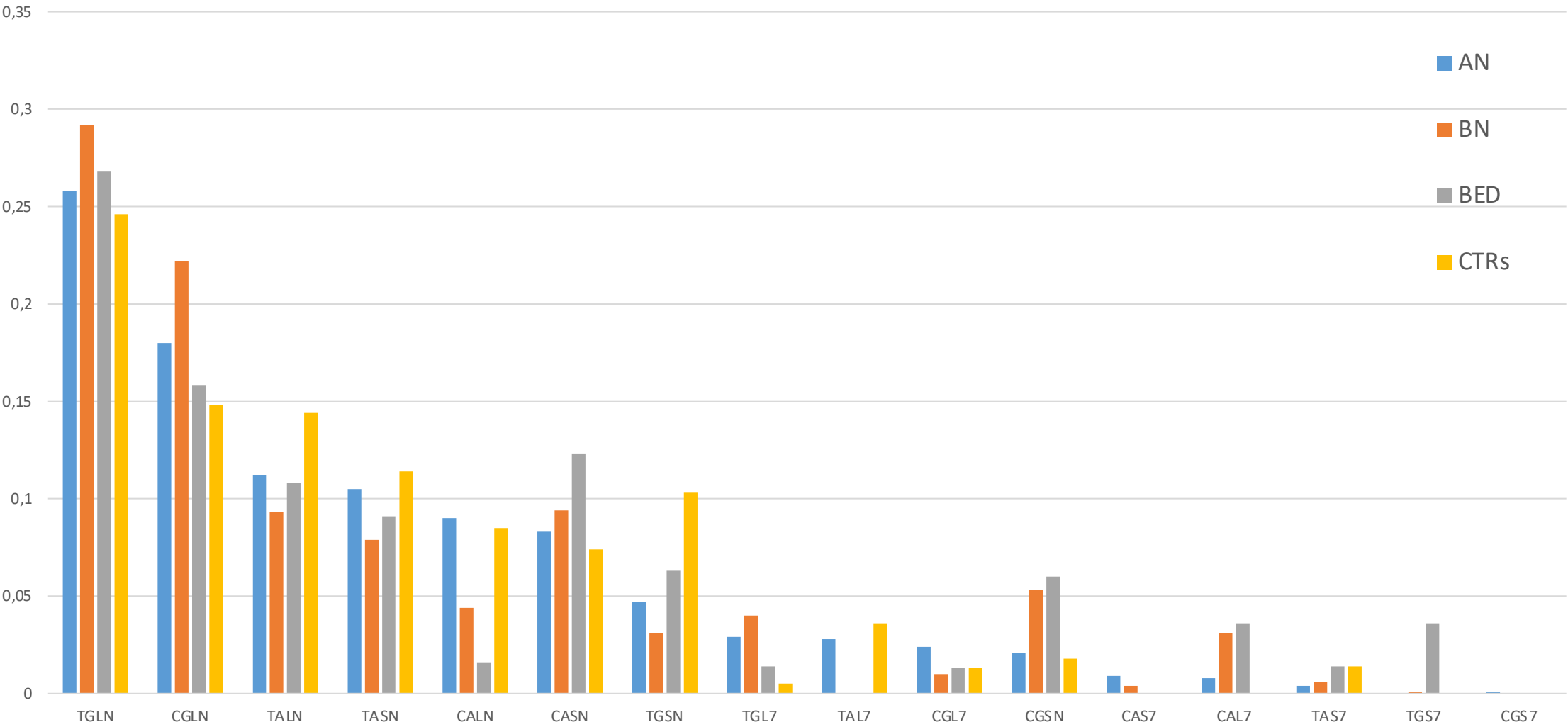

Supplement: Supplementary file 2 [file Image_1.pdf]
